# Addison's Disease and Possible Cannabis Withdrawal Syndrome Presenting as an Eating Disorder in a Thirty-Year-Old Female

**DOI:** 10.1155/2017/4096021

**Published:** 2017-03-01

**Authors:** Kimberly Lazare

**Affiliations:** Department of Family and Community Medicine, North York General Hospital, University of Toronto, Toronto, ON, Canada

## Abstract

A 30-year-old female with a history of anxiety, cannabis use, and Avoidant/Restrictive Food Intake Disorder presented for residential treatment of a Cannabis Use Disorder. Upon arrival, she had not eaten for two days and was found to be hypotensive with electrolyte disturbances. She was admitted to a nearby hospital, where the Internist diagnosed her with Addison's disease. She was treated with corticosteroid therapy, with rapid normalization of her electrolytes, eating, and anxiety. This is the first published case of undiagnosed Addison's disease presenting as an eating disorder, with cannabis use likely contributing to symptoms. This case elucidates the importance of ruling out other biologic and psychologic causes of clinical presentations before an eating disorder diagnosis can be made.

## 1. Introduction

Eating disorders are psychiatric disorders predominantly affecting female adolescents and young adults [1]. They are characterized by body image concerns, disordered eating, and in some cases, such as with Anorexia Nervosa, refusal to maintain a normal weight [2]. In severe cases, electrolyte abnormalities and ECG changes can lead to hypotension, syncope, and even death [3]. Due to eating disorders having relatively low prevalence and with a generalized lack of training in assessment, diagnosis, and treatment of eating disorders for clinicians, eating disorders often get misdiagnosed or missed altogether. If there is clinical suspicion for an eating disorder, one must first exclude other organic causes to explain the patients' symptoms, including Addison's disease, a rare endocrine disorder resulting from deficiency of adrenocortical hormones [4]. Other psychiatric causes of disordered eating, such as Cannabis Withdrawal Syndrome (CWS), must also be excluded [5]. Hasty assumptions of diagnosis can have potentially serious consequences.

## 2. Case Report

An emaciated 30-year-old female arrived at a residential addiction treatment centre for a six-week program for treatment of a Cannabis Use Disorder. She endorsed escalating use of marijuana in the last few years and estimated smoking one gram per day in recent months. When she first started using marijuana in her early twenties, she only used it socially with friends. She reported that her use escalated in the last few years specifically to control nausea and to increase her appetite. In fact, she could not eat a meal in the last year without first smoking marijuana. She had also been experiencing low mood, anxiety, and panic attacks for the past six years since starting graduate school, for which she was on Cipralex 20 mg daily and Wellbutrin XL 300 mg daily. She endorsed vomiting a few times per week to once daily. This vomiting was not in response to feelings of guilt after having eaten too much but rather a response to feelings in her stomach when she had not eaten enough or when she had eaten without marijuana use. She denied any body image issues. She had lost twenty pounds over the past year, with a current weight of 49.6 kg and a BMI of 17. She had been diagnosed by her Psychologist with Avoidant/Restrictive Food Intake Disorder and had been in outpatient therapy for her eating disorder for the past year. Other aspects of medical history included a diagnosis of hypothyroidism, for which she was taking thyroid hormone replacement.

Upon arrival at the residential facility, she endorsed having not eaten anything for at least the past two days and was feeling lightheaded and unwell. Her blood pressure measured 80/49. She was subsequently sent to a nearby hospital for assessment. In the Emergency Department, her blood pressure was 88/54. Serum sodium was 126 mmol/l, potassium was 4.8 mmol/l, urea was 8.9 mmol/l, creatinine was 97 mmol/l, and glucose was 3.1 mmol/L. As a result of her symptoms and hyponatremia, she was admitted to the Internal Medicine service. The Endocrinologist who saw her during her admission noticed that her skin had a bronzed hue, and the patient shared that her skin suddenly became tanned nine months earlier. Her potassium level rose to 6.3 mmol/l the following day. Given her symptoms, hyperpigmentation, and lab findings, a presumptive diagnosis of Addison's disease was made. ACTH was measured to be >278 mmol/L, and random cortisol was 25 mmol/L. She was started on Hydrocortisone 10 mg daily and discharged back to the residential facility once her symptoms and lab abnormalities normalized. Within a few days, her eating completely normalized, she denied any nausea or vomiting, and her anxiety resolved. She went on to successfully complete the residential program.

## 3. Discussion

There are only a handful of published case reports of patients misdiagnosed with an eating disorder, most commonly Anorexia Nervosa (AN), who actually had underlying Addison's disease [6–11]. There are also only a handful of cases of patients with CWS misdiagnosed with an eating disorder [5]. Our case represents the first published case of a patient with undiagnosed Addison's disease and possible CWS who was misdiagnosed as having an eating disorder.

In our patient, there were several features of the medical history which made the diagnosis of an eating disorder unlikely. Our patient was not obsessed with weight and shape and in fact felt the opposite; she felt that she was too thin and needed to gain weight. Her food preferences included a number of high-fat foods, such as pizza and chili. Her bloodwork showed hyperkalemia, hypoglycemia, and hypocortisolism, all lab features of Addison's disease, as compared to the hypercortisolism, hyperglycemia, and hypokalemia seen with eating disorders such as AN [12]. Bronzed skin is a pathognomonic feature of Addison's disease and should have skewed her doctors towards an alternate diagnosis.

In contrast, weight loss, hyponatremia, nausea and vomiting, fatigue, orthostatic hypotension, and ECG changes are features of both restrictive eating disorders such as AN and Addison's disease, making them difficult to distinguish clinically [13]. Several behavioural similarities are also observed; eating disorder patients often suffer from depression and blunted emotional expression, which can be similar to the behavioural complaints seen in chronic adrenal insufficiency such as poor concentration, irritability, and depression [14]. Thus, for the Psychiatrist or Psychologist who have little to no Internal Medicine training, one can see how she mistakenly received an eating disorder diagnosis in this case.

CWS is another important diagnostic consideration to consider in heavy cannabis users presenting with gastrointestinal (GI) complaints, disordered eating, and weight loss. DSM-V diagnostic criteria for CWS include at least three of the following symptoms:

1. Irritability, anger, or aggression
2. Nervousness or anxiety
3. Sleep difficulty (insomnia)
4. Decreased appetite or weight loss
5. Restlessness
6. Depressed mood
7. Physical symptoms causing significant discomfort from at least one of the following: stomach pain, shakiness/tremors, sweating, fever, chills, or headache

These symptoms must cause significant distress or impairment in social, occupational, or other important areas of functioning, and the symptoms must not be due to a general medical condition and must not be better accounted for by another disorder [2].

Due to the diagnostic criteria for CWS stipulating that the symptoms must not be due to a general medical condition, we cannot say for certain that our patient had a diagnosis of CWS given that her symptoms were explained by the undiagnosed Addison's disease. However, a number of features of our patient's case are markedly similar to other cases of CWS; in an article by Chesney et al. [5], they presented a case of CWS in a 16-year-old female with daily nausea, vomiting, decreased appetite, and abdominal pain. Their case reported using self-induced vomiting and cannabis for relief of abdominal pain, similar to our patient. She denied vomiting for the purpose of weight loss and felt she was too thin, having lost 20 pounds unintentionally over the past three months. Her mornings were characterized with feelings of panic in association with her GI symptoms, similar to our patient. Our patient endorsed feeling physically better after using marijuana consistently throughout the day, with symptoms being worst in the morning after waking from sleep after several hours of abstinence from cannabis use. This fits with research indicating that withdrawal symptoms are most severe on day one of abstinence [15].

Even though we cannot say with absolute certainty that our patient met diagnostic criteria for CWS, marijuana did play a significant role in her day-to-day life, and her use escalated in recent years to help control GI symptoms. One wonders what role cannabis would have played in her life if Addison's disease had been diagnosed earlier on.

Another issue not readily discussed in other case reports is that once a patient receives a diagnosis of an eating disorder, it is difficult for that label to be contested. There are still a lot of stigmata surrounding an eating disorder diagnosis; these patients are known for their secrecy and it is common for clinicians to mistrust their reports and attribute all their physical symptoms to their underlying psychiatric illness(es). This was true for our patient even after she received a diagnosis of Addison's disease as the Internist who diagnosed her with Addison's disease still listed “eating disorder” as a diagnosis on her hospital discharge summary.

## 4. Conclusions

This case report raises a number of important issues that clinicians can learn from. Clinicians must conduct a thorough medical and psychiatric evaluation before a diagnosis of an eating disorder can be made. Improved cross-training across disciplines (i.e., educating Internists about eating disorders and educating Psychologists and Psychiatrists about Addison's disease) needs to be considered. Given that CWS is a relatively new diagnosis, further research needs to be conducted to gain a better understanding of the role that cannabis plays in these complex disorders. Finally, we must continue to battle the stigma that eating disorders have in the medical profession to ensure that these patients receive the same standard of care as all other patients presenting with serious symptomatology.
